# Supplementary figures and images for: Dissolved hydrogen and nitrogen fixation in the oligotrophic North Pacific Subtropical Gyre
Source: Environ Microbiol Rep. 2013 Jun 10;5(5):697–704. doi: 10.1111/1758-2229.12069 (PMC4809409; doi:10.1111/1758-2229.12069)

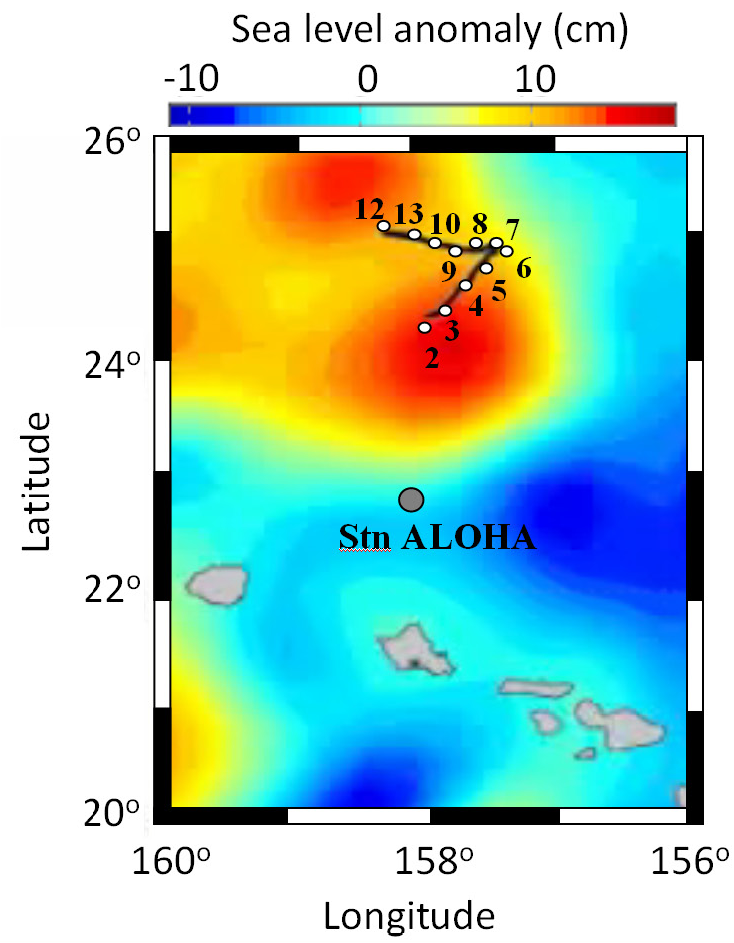

Supplement: Supplementary file 2 — Figure S1. 14‐day composite of satellite derived SSHA 100 km north of the Hawaiian Islands in the Pacific Ocean between 7 and 21 September 2011 (data from Moderate Resolution Imaging Spectroradiometer). A summary of the cruise transect is indicated by the solid black line and the labeled white circles represent the sampling stations discussed in the text. Station ALOHA, the long‐term sampling station for the Hawaii Ocean Time‐series (HOT) programme, located at 22°45′N, 158°W is also highlighted. [file EMI4-5-697-s001.tif]

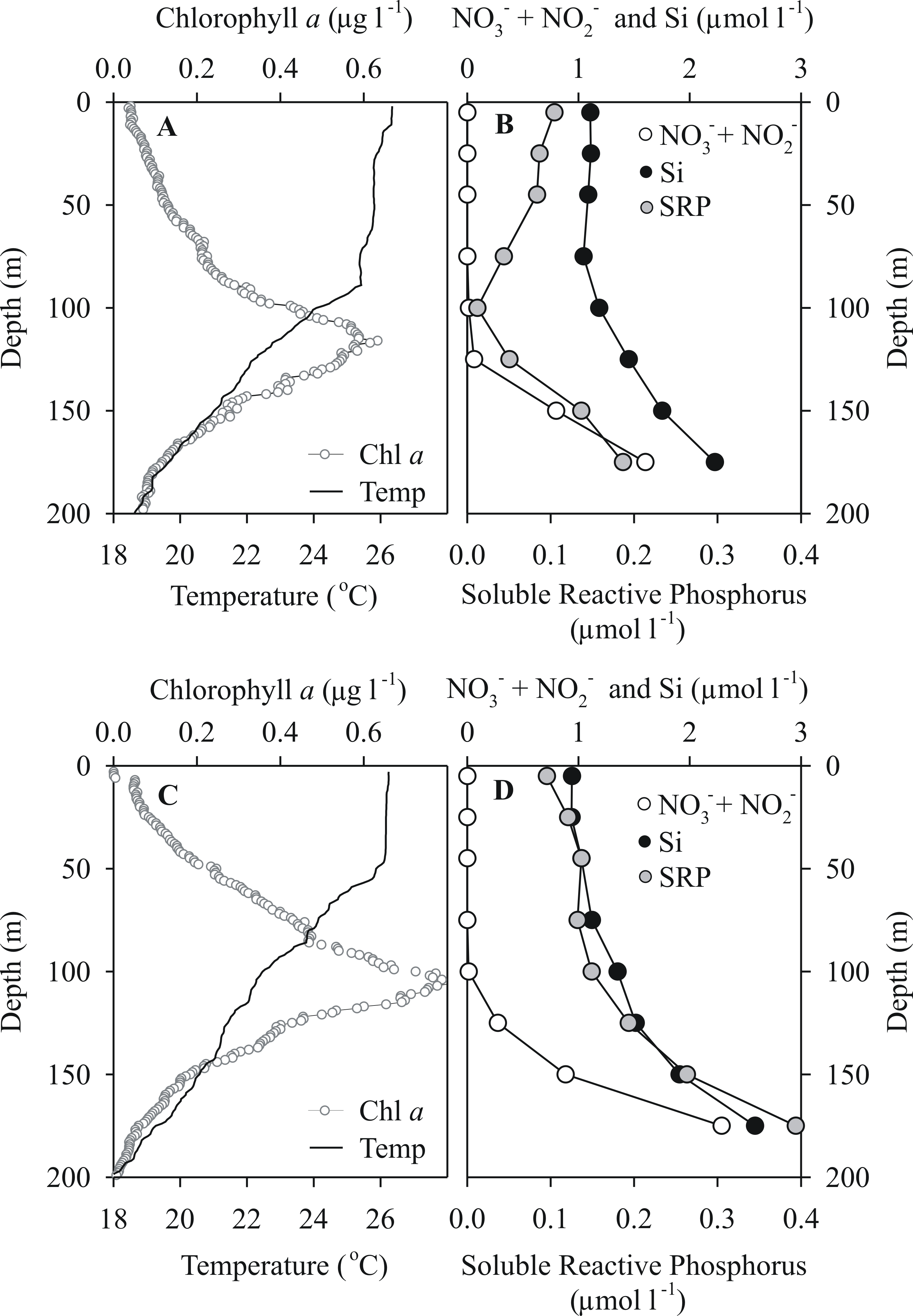

Supplement: Supplementary file 3 — Figure S2. Representative water column profiles for the two sections of the cruise track, (A‐B) Stn 3 and (C‐D) Stn 13. [file EMI4-5-697-s002.tif]
